# Intraoperative near-infrared imaging can identify canine mammary tumors, a spontaneously occurring, large animal model of human breast cancer

**DOI:** 10.1371/journal.pone.0234791

**Published:** 2020-06-17

**Authors:** Andrew Newton, Jarrod Predina, Michael Mison, Jeffrey Runge, Charles Bradley, Darko Stefanovski, Sunil Singhal, David Holt

**Affiliations:** 1 Department of Surgery, University of Pennsylvania Perelman School of Medicine, Philadelphia, Pennsylvania, United States of America; 2 Department of Clinical Sciences and Advanced Medicine, University of Pennsylvania School of Veterinary Medicine, Philadelphia, Pennsylvania, United States of America; 3 Department of Pathobiology, University of Pennsylvania School of Veterinary Medicine, Philadelphia, Pennsylvania, United States of America; 4 Department of Clinical Studies New Bolton Center, University of Pennsylvania School of Veterinary Medicine, Kennett Square, Pennsylvania, United States of America; Colorado State University, UNITED STATES

## Abstract

**Introduction:**

Current methods of intraoperative margin assessment in breast conserving surgery are impractical, unreliable, or time consuming. We hypothesized that intraoperative near-infrared (NIR) imaging with an FDA-approved NIR optical contrast agent could identify canine mammary tumors, a spontaneous large animal model of human breast cancer, during surgery.

**Methods:**

Dogs with mammary tumors underwent a standard of care lumpectomy or mastectomy with wide surgical margins 20 hours after indocyanine green administration (3 mg/kg IV). During surgery, NIR imaging was performed on tumors and wound margins *in situ* and tumors and lymph nodes *ex vivo*. Following resection, the wound bed was examined for residual fluorescence. Fluorescence intensity was determined by signal-to-background ratio (SBR). All tumors, areas of residual fluorescence, and lymph nodes underwent histopathologic analysis.

**Results:**

There were 41 mammary tumors in 16 female dogs. Twenty tumors were malignant and 21 were benign. Twenty-eight tumors were fluorescent (mean SBR 1.5±0.2). Sensitivity of fluorescence for all malignant tumors was 80% (16/20) and 93.3% (14/15) for malignant tumors > 2 cm. Specificity for malignancy was low (< 2cm = 55%; > 2cm = 30%). Tumors > 2 cm were more likely to be fluorescent (OR 6.05, 95% CI 1.50–24.44, P = 0.011) but not more likely to be malignant (OR 3.09, 95% CI 0.86–11.14, P = 0.085) than tumors ≤ 2 cm. Four out of seven inguinal lymph nodes excised in the mastectomy specimen fluoresced. All four drained malignant tumors; however only 2/4 contained metastatic disease.

**Conclusion:**

Systemic ICG accumulates reliably in malignant canine mammary tumors > 2 cm. Although no tumor margins fluoresced, a wider margin of normal tissue is removed in canine mastectomy, making direct comparisons with breast conserving surgery difficult. Targeted NIR imaging agents are likely required to improve detection of smaller tumors and improve the specificity of NIR imaging for residual disease and metastatic lymph node detection.

## Introduction

Breast cancer is the second most common cause of cancer death and the most common cancer overall in women with over 250,000 estimated new cases in the United States in 2017 [1]. The widespread use of screening mammography has increased detection of small tumors, and as a consequence, breast-conserving surgery (BCS) is now used more commonly [2]. The aim of BCS is complete tumor resection to minimize local recurrence while retaining enough breast tissue for an acceptable cosmetic result. Radiation is required after BCS, and large, prospective, randomized clinical trials have shown this multimodality therapy results in equivalent survival compared to modified radical mastectomy [3–5]. Despite adjuvant radiation, ipsilateral breast cancer recurrence still takes place in 6–9% of patients following BCS for invasive breast cancer, while ipsilateral recurrence rates following BCS for ductal carcinoma in situ (DCIS) are even higher [6,7].

Multifocal disease, invasive disease, carcinoma in situ, or a combination of these conditions complicates surgery and may result in incomplete tumor excision [8]. Positive margins on the BCS specimen are associated with local recurrence [9,10], and these patients usually undergo a second surgery for re-excision. While re-excision rates vary widely by institution [8,9], large series suggest re-excision rates are approximately 13–23% [8,10–12]. Re-excision requires additional anesthesia, increases the risk of complications including surgical site infection, and incurs increased cost [13,14]. In an effort to decrease local recurrences and re-excision rates, several approaches to improve intraoperative tumor margin assessment are currently used, including intraoperative ultrasound, intraoperative specimen mammography, radioactive seed localization, radiofrequency spectroscopy, and frozen section pathology with or without imprint cytology [15–20]. However, these techniques require operator expertise, can involve radiation exposure, are impractical, or are time consuming.

The risk of positive margins requiring re-excision in breast cancer clearly indicates the need for better intraoperative evaluation of surgical margins. Intraoperative NIR imaging with systemically injected NIR contrast agents is a novel method for tumor localization and margin detection that is being investigated in a variety of solid malignancies [21]. With an appropriate contrast agent, NIR imaging could potentially identify the tumor, tumor margins, and sentinel lymph nodes. Sentinel lymph nodes in human breast cancer are most commonly identified with the combination of a radioactive tracer and a blue dye [22], although some groups now use intraoperative near-infrared (NIR) imaging with indocyanine green (ICG) [23]. In all current sentinel lymph node identification techniques, lymph nodes are visualized after a peritumoral or subareolar injection of dye. It is not clear if systemically administered NIR contrast agents can identify sentinel lymph nodes in either canine mammary tumors or human breast cancer.

ICG is the only U.S. Food and Drug Administration (FDA)-approved contrast agent used for NIR imaging. We have previously shown in experimental small animal and translational, large animal solid tumor models that a high dose of intravenous ICG will accumulate preferentially in tumors over 24 hours by the enhanced permeability and retention (EPR) effect [24–26]. After dye accumulation, NIR imaging can be used to identify the tumor, tumor margins, and locate residual disease in spontaneously occurring canine lung cancers and sarcomas [25,26]. Canine mammary tumors are an excellent clinical and molecular model for human breast cancer [27–31]. There has been limited evaluation of NIR imaging in canine mammary tumors as models of human breast cancer [32–34]. In this study, we hypothesized that NIR imaging with systemic ICG could detect canine mammary tumors and metastatic lymph nodes in a spontaneously occurring, large animal model of human breast cancer.

## Materials and methods

### Canine subjects

Dogs were recruited through the clinic at the Matthew J. Ryan Small Animal Hospital at the University of Pennsylvania School of Veterinary Medicine and the School’s Canine Shelter Mammary Tumor program. Eligible dogs had mammary tumors on physical examination, had no evidence of metastatic disease on thoracic radiographs, and had no substantial comorbidities that would impair their ability to tolerate anesthesia and surgery. The study was approved by the University of Pennsylvania Institutional Animal Care and Use Committee. Written informed consent was obtained from all owners. All dogs were treated as clinical patients of the Matthew J. Ryan Small Animal Hospital with clinical standard of care housing, feeding and watering, pain management, and nursing.

### Study drug

Pharmaceutical grade indocyanine green (ICG) (3 mg/kg) (Akorn, Lake Forest, IL, product number NDC 17478-701-02) was administered intravenously over 3 minutes 20 hours prior to surgery. ICG is a NIR contrast agent with peak excitation and emission of 805 nm and 830 nm, respectively. This dose was chosen based on our previous experience with other spontaneous solid tumors in dogs [25,26].

### Imaging system

The surgical field was imaged with a prototype imaging system (Solaris, Perkin Elmer). Fluorescence excitation was generated by four banks of LED lights (two white lights and four excitation lights each, 2W, 750 nm peak wavelength; 730–750 nm bandpass excitation filter). Emitted light was collected through an optical system containing a 760-841nm bandpass filter. The system had a 10 cm field of view and a working distance of 75 cm. Emitted light was captured by two scientific complementary metal oxide semiconductor (sCMOS) cameras, one for fluorescent light and one for visible light. The system employed a pulsing scheme to prevent ambient light from biasing the fluorescent image. A foreground frame was captured with the excitation LED lights on and a subsequent background frame was acquired with the LED lights off. A 10 ms exposure time was used for both images. The background frame was then subtracted from the foreground frame and the image displayed on a computer screen.

### Study design

Under general anesthesia, a standard-of-care lumpectomy or mastectomy was performed at the discretion of the surgeon. As these were clinical cases, all tumors were excised with a wide margin (1–2 cm) of grossly normal surrounding tissue. Excisions were performed to the level of the fascial sheath of the rectus abdominus muscles.

During surgery, the tumor and wound edges were evaluated for fluorescence. Following excision, the wound bed and surrounding normal tissue was imaged once again with the fluorescence imaging system. Any fluorescence (assigned the color green by the imaging system) visible to the surgeon was considered a positive result. One surgeon performed all evaluations for fluorescence. Samples of areas of residual fluorescence were surgically excised and submitted for histopathological evaluation. Tumors and surrounding excised tissues were imaged *ex vivo*, and margins of the excised specimens were evaluated for fluorescence. Margins of the specimens were inked and submitted for histopathological evaluation. The presence or absence of fluorescence at the resection margins was compared to histopathologic margin status. Excised specimens involving the caudal mammary gland(s) were imaged to evaluate for inguinal lymph node fluorescence. Lymph nodes were then dissected from the excised specimens, imaged *ex vivo*, and submitted for histopathological evaluation.

The NIR fluorescence of tumors and normal tissue was quantified using the region of interest (ROI) plugin of ImageJ^®^ (National Institutes of Health; http://rsb.info.nih.gov/ij/) and compared as signal-to-background ratios (SBR). The range, mean, and standard deviation of fluorescence intensity within each specific region of interest were obtained using the ImageJ^®^ histogram function. The “signal” was quantified as the average fluorescence from the regions identified as tumor. The “background” was identified as the average fluorescence of adjacent normal mammary tissue. Fluorescence of identified lymph nodes was compared to the highest fluorescence value for surrounding normal tissue (adipose tissue) in that dog.

### Statistical analysis

All statistical analysis was performed using STATA 15 (StataCorp, College Station, TX). Descriptive statistics are presented as median and range. The sensitivity of ICG for detecting malignancy was defined as the number of fluorescent malignant tumors divided by the total number of malignant tumors. The specificity of ICG for detecting malignancy was defined as the number of non-fluorescent benign tumors divided by the total number of benign tumors. Pearson’s chi square test was used to examine the association of tumor with malignancy and fluorescence. Firth penalized maximum likelihood logistic regression was also used to assess the above-mentioned association. Firth logistic was chosen due to a problem of “perfect prediction.” Perfect prediction is encountered when the outcome variable perfectly differentiates a predictor variable or variables. Linear regression was used to quantify the association between tumor fluorescence and tumors grouped by size. Post-hoc pairwise comparison was used to identify differences between the groups at the level of P = 0.05.

## Results

### Subject characteristics

Sixteen dogs were enrolled in this study. Ages ranged from 5–15 years (Median = 9 years); the age of one dog was unknown. One dog had a second surgery for mammary carcinoma recurrence and imaging was performed during both procedures. All dogs were female; 9/16 were intact, and the other 7 were spayed. Weights ranged from 2.1–39.5 kg (Median = 17.3 kg). Table 1 has full subject characteristics.

**Table 1:** Characteristics of study subjects.

Case	Age	Breed	Weight (kg)	Intact	Mammary Glands Involved	Malignant Tumors: Number, Size (cm)	Benign Tumors: Number, Size (cm)
**1**	11	Mixed	4.2	Yes	L4, R4, R5	0: n/a	3: 0.5–2
**2**	9	Golden Retriever	30.0	Yes	L2	0: n/a	1: 2
**3a**	10	Mixed	17.3	Yes	L5	1: 2	0: n/a
3b*					L5, R5	2: 3,4	0: n/a
**4**	7	German Shepherd	35.0	No	R2, R3, R4, R5, L3	1: 5	4: 0.2–4
**5**	Unknown	Mixed	3.5	No	R2, R3, R4, R5	1: 1	3: 0.2–2
**6**	7	Chihuahua	4.6	Yes	R4, L4	0: n/a	2: 2,2
**7**	11	Chihuahua	2.1	No	L4, L5	0: n/a	2,:0.5, 1
**8**	14	Lhasa Apso	10.8	No	L3, L4, L5	2: 5, 5	1: 0.5
**9**	9	Cocker Spaniel	8.5	No	R4	1: 2	0: n/a
**10**	13	Chihuahua	2.6	Yes	R1, R2, R3, R4, R5, L2, L3, L4, L5	6: 1–2	3: 0.5, 0.5,0.5
**11**	14	Border Collie	18.0	Yes	R5	1: 3	0: n/a
**12**	9	Bulldog	25.0	No	L3	1: 3	0: n/a
**13**	15	Mixed	17.8	No	L2, L4	0: n/a	2: 0.5, 2
**14**	6	Boston Terrier	16.7	Yes	R3	1: 8	0: n/a
**15**	8	German Shepherd	39.5	Yes	R5	1: 4	0: n/a
**16**	12	Saluki	29.5	Yes	R3, R5	2: 3, 8	0: n/a

Abbreviations: L: left, R: right; n/a: Not applicable

* A second surgery was performed on Case #3 for mammary tumor recurrence

### Intraoperative near-infrared imaging identifies mammary tumors in vivo

**Fig 1:**
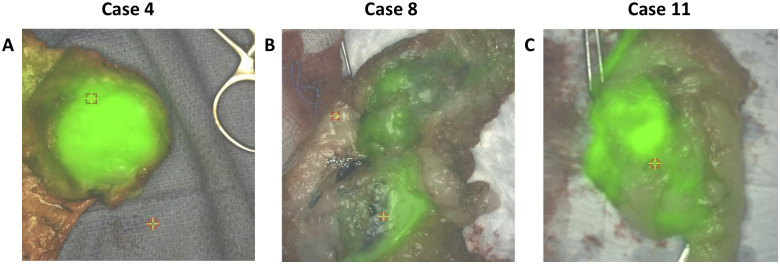
Representative intraoperative images of fluorescent malignant tumors. A) 5 cm papillary adenoma with low grade ductal adenocarcinoma. B) 7 cm intermediate grade carcinoma. C) 3 cm low grade carcinoma. In this case, one of the non-fluorescent margins is obscured by specimen positioning.

The lowest SBR at which the surgeon could perceive visible fluorescence intraoperatively corresponded with an SBR = 1.2 and all tumors with a SBR ≥ 1.2 fluoresced. A total of 41 tumors were imaged; 28 were fluorescent (mean SBR = 1.5±0.2). Representative cases are shown in Fig 1. Fluorescence was not seen beyond the margins of any of the tumor specimens and there were no tumor-positive margins on histopathology.

Tumor fluorescence and fluorescence sensitivity, specificity, and positive and negative predictive values for malignancy stratified by tumor size and histopathology (benign vs malignant) are summarized in Table 2. The overall sensitivity of NIR imaging for detection of all malignant tumors was 80% (16/20). The sensitivity of NIR imaging for detection of malignant tumors > 2 cm was 93.3% (14/15). Specificity of fluorescence for malignancy was 55% for tumors < 2cm and 30% for tumors >2cm. Tumors > 2 cm were more likely to be fluorescent (OR 6.05, 95% CI 1.50–24.44, P = 0.011) but not more likely to be malignant (OR 3.09, 95% CI 0.86–11.14, P = 0.085) than tumors ≤ 2 cm.

**Table 2:** Tumor fluorescence and fluorescence sensitivity, specificity, and positive and negative predictive values for malignancy stratified by tumor size and histopathology (benign vs malignant).

Tumor Size	Malignant N (SBR Range)	Benign N (SBR Range)	Sensitivity	Specificity	PPV	NPV
**0–2 cm**			40%	54.5%	0.29	0.67
Fluorescent	2 (1.6)	5 (1.1–1.4)				
Non-Fluorescent	3 (0.9–1.0)	6 (0.9–1.1)				
**2.1–5 cm**			90.9%	30.0%	0.59	0.3
Fluorescent	10 (1.4–2.1)	7 (1.2–1.8)				
Non-Fluorescent	1* (1.0)	3 (0.8–1.1)				
**>5 cm**			100.0%	N/A	1.0	N/A
Fluorescent	4 (1.3–1.7)	0				
Non-Fluorescent	0	0				

* One tumor malignant tumor that did not fluoresce was surrounded by a dense fibrous capsule

N = number; SBR = signal to background ratio

PPV = Positive predictive value

NPV = Negative predictive value

### Intraoperative near-infrared imaging of the wound bed identifies additional disease but also inflammation and normal tissue

**Fig 2:**
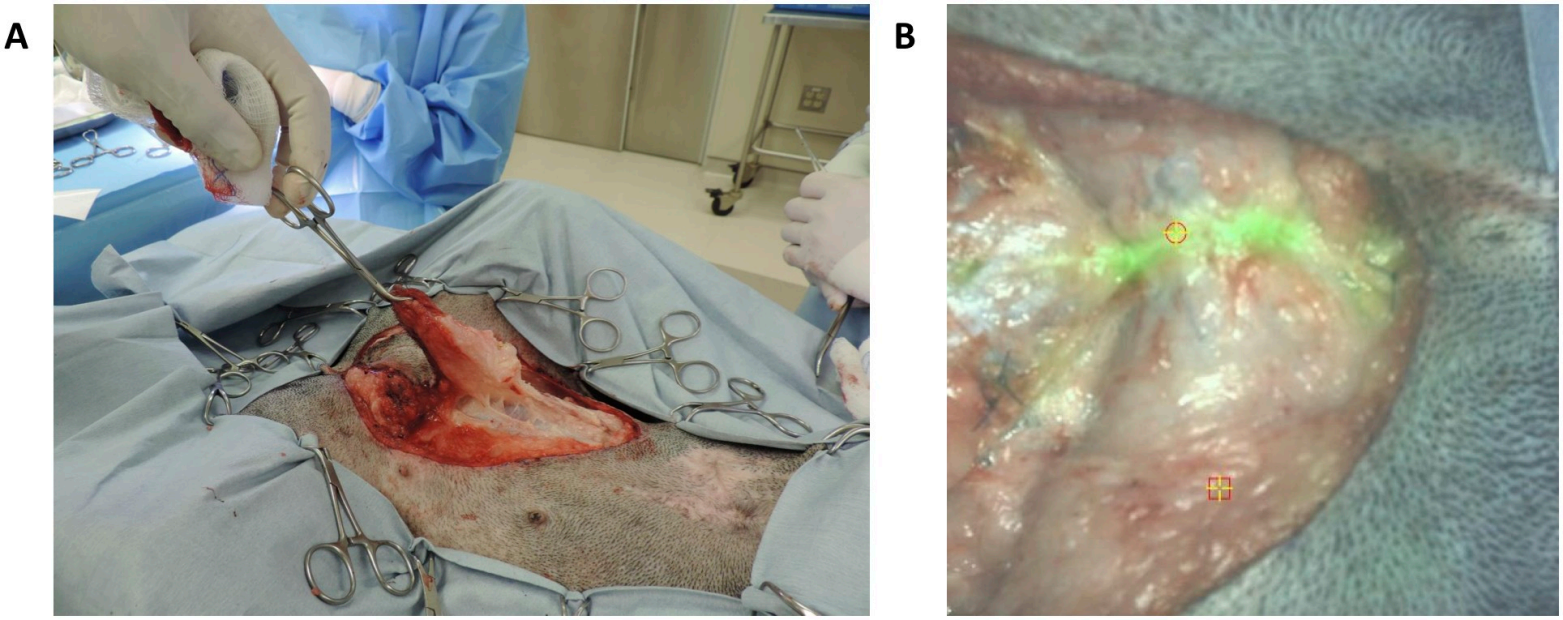
Intraoperative images from case 15. A) The lumpectomy specimen is being elevated from the operative field. The primary tumor was surrounded by dense connective tissue and was not fluorescent. B) Fluorescence was seen in the wound bed following resection of the primary tumor. Biopsy of this area demonstrated mammary carcinoma.

Five cases (4 dogs) had visible fluorescence in the wound bed following tumor resection. One of these fluorescent areas represented an additional focus of mammary carcinoma. In this dog, the primary tumor, which was surrounded by a dense layer of connective tissue (Fig 2A), was not fluorescent. The primary tumor was resected via lumpectomy. The tumor was not sectioned to assess fluorescence within the tumor parenchyma proper. After the surgeons felt they had performed a complete resection, the wound bed was examined, and an additional area of fluorescence (SBR 2.2) was seen (Fig 2B). This area was biopsied, and final pathology revealed mammary carcinoma in both the initial lumpectomy and residual fluorescence specimens. The four remaining cases had no residual neoplastic disease in the wound bed in spite of wound bed fluorescence (Table 3).

**Table 3:** Results of NIR imaging and histopathology in subjects with residual wound bed fluorescence.

Case Number	Maximum SBR of Primary Tumors	Final Pathologies of Primary Tumors	SBR of Residual Fluorescence	Final Pathology of Additional Biopsy
**1**	1.2	Adenoma Complex adenoma Ductal adenoma	1.3	Normal muscle
**3a**	1.5	Mammary carcinoma	1.6	Lobular hyperplasia
**3b**	1.8	Mammary carcinoma	1.5	Suture granuloma
**10**	1.6	Complex adenoma	1.5	Normal linea
**15**	1.0	Mammary carcinoma	2.2	Mammary carcinoma

Abbreviations: SBR: signal-to-background ratio

### Intraoperative near-infrared imaging using systemic icg identifies lymph nodes but does not discriminate benign from malignant nodes

**Fig 3:**
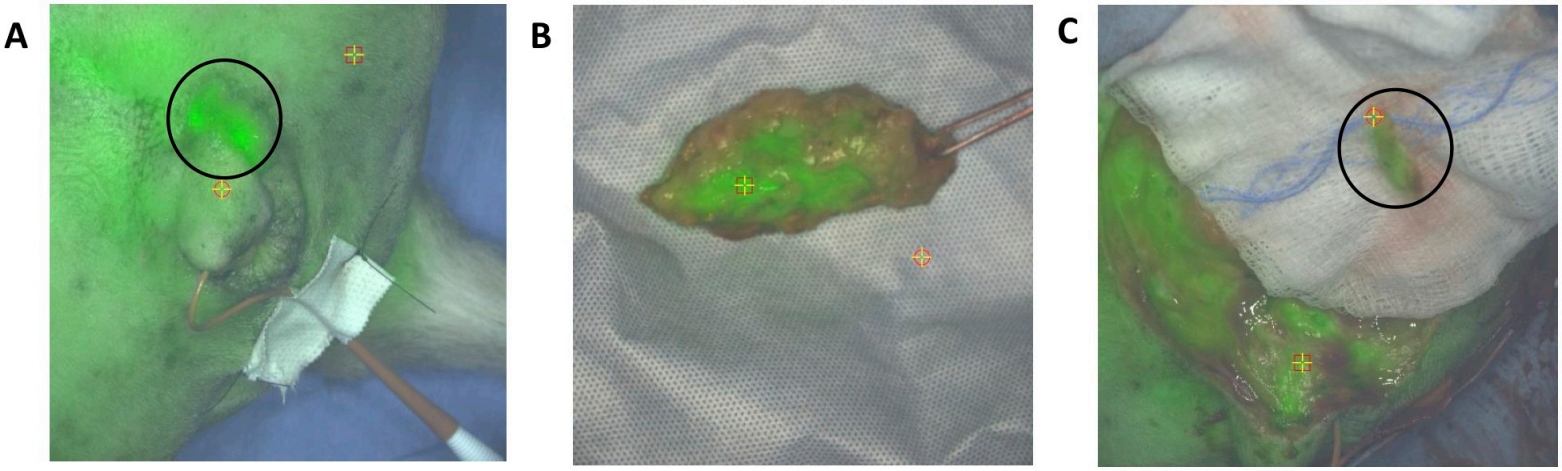
Intraoperative images from case 3b. A) Tumor fluorescence could be seen through the skin prior to any dissection (circle). B) 2 cm mammary carcinoma on the back table following resection. C) Fluorescent lymph node with metastatic disease (circle). The background fluorescence was associated with suture granulomas and inflammation associated with the previous surgery.

Lymph nodes were visualized in the resected specimen in 7 dogs. Two of these dogs had benign tumors and 5 had malignant tumors. Lymph nodes were surrounded by mammary tissue and fat and not immediately fluorescent in the resected specimen. Lymph nodes were all visible with white light once the surrounding tissue was dissected; four out of seven were fluorescent on NIR imaging (median SBR = 1.6, range 1.3–1.7). All fluorescent lymph nodes were associated with fluorescent tumors. Two out of four fluorescent lymph nodes were positive for malignancy while the other two fluorescent lymph nodes showed drainage reaction on histopathology. In case 3a, both the tumor (Fig 3A and 3B) and malignant lymph node (Fig 3C) were fluorescent. Pathology demonstrated drainage reaction in the three non-fluorescent lymph nodes. These results are summarized in Table 4.

**Table 4:** Summary of tumor and lymph node fluorescence for specimens with inguinal lymph nodes identified.

Case	Mammary Glands Involved	Tumor Size (cm)	Maximum Tumor SBR	Tumor Pathology	LN SBR	Final Histopathology
**3b**	L5, R5	3, 4	1.8	Mammary carcinoma	1.6	Mammary carcinoma
**4**	R2, R3, R4, R5, L3	0.2, 4, 5, 4, 3	1.5	Adenoma (R2), Ductal carcinoma (R4)	1.3	Drainage reaction*
**7**	L3, L4	0.5, 1	1.4	Complex adenoma	1: NF	Drainage reaction*
**12**	L3	3	1.6	Mammary carcinoma	1.7	Drainage reaction*
**13**	L2, L4	0.5, 2	1.2	Lobular hyperplasia, complex adenoma	1: NF	Drainage reaction*
**15**	R5	4	1.0	Mammary carcinoma	1.6	Mammary carcinoma
**16**	R3, R5	3, 8	1.7	Mammary carcinoma	1: NF	Drainage reaction*

Abbreviations: SBR signal-to-background ratio, LN lymph node, L left, R right, NF non-fluorescent

* “Drainage reaction”: Mild lymphoid hyperplasia with increased numbers of histiocytes, plasma cells, and erythrocytes in the subcapsular trabeculae and medullary sinuses.

## Discussion

The goal of intraoperative imaging during BCS is to ensure complete, tumor-negative surgical margins to minimize the risk of local recurrence. In this cohort of dogs, fluorescence was not seen at the resection margins in any case and there were no corresponding tumor-positive margins on histopathology. The gross margins removed in these dogs were likely larger than those taken in BCS as this is standard of care in canines, making fluorescent and tumor-positive margins less likely. Although canine mammary tumors have many clinical and molecular similarities to human breast cancer [27–31], the standard of care resection for canine mammary tumors clearly limits the utility of these dogs as a model for margin assessment in human breast conserving surgery. In a previous study, ICG tumor fluorescence extended 3 mm beyond the actual tumor in canine sarcomas [26]. Should the same occur using ICG in human breast cancer, intraoperative imaging with ICG would result in some overestimation of the extent of disease and may increase the margin of normal breast tissue removed when performing tumor resection.

Not all tumors fluoresced, particularly those <2 cm diameter. This represents another possible limitation of this approach, as tumors resected in breast conserving surgery in human patients are often 2 cm diameter or less [35]. The lack of fluorescence in smaller benign tumors may have occurred because of a lack of tumor neovasculature required for ICG accumulation via the EPR effect. In more aggressive tumors, angiogenesis produces a vasculature with a defective endothelial cell lining, allowing ICG to leak into the tumor preferentially [36,37] and accumulate due to properties including size, shape, charge, polarity [38] and a lack of normal tumor lymphatic drainage. ICG acts a as a macromolecule in this instance due to albumin binding in the circulation [39]. Decreased tumor microvessel density has been associated with decreased expression of hypoxia-inducible factor in more benign human breast cancers [40]. The lack of fluorescence in some smaller malignant lesions is more problematic. Several of these tumors did not accumulate enough ICG to fluoresce during surgery. ICG needs to be present in concentrations above micromolar for interoperative detection [41].

Although this study was not focused on using intraoperative imaging to differentiate malignant from benign disease, NIR imaging had a 93% sensitivity for the detection of malignant canine mammary tumors > 2 cm diameter. The one larger tumor that did not fluoresce was surrounded by a dense layer of desmoplastic tissue. It is possible this dense connective tissue prevented passage of the excitation and emission NIR light wavelengths required for fluorescence imaging. However, the specificity of NIR imaging for malignancy was low and decreased with increasing tumor size (Specificity < 2cm = 55%; specificity > 2cm = 30%). Fluorescence rates of benign mammary tumors increased with size (Table 2). This is not surprising considering the mechanism of ICG accumulation. Although malignant canine mammary tumors show significantly greater angiogenesis than benign tumors [42], both malignant and benign mammary tumors have increased intra- and peritumoral blood vessel density and endothelial cell proliferation compared to normal mammary tissue [43].

Residual wound bed fluorescence was seen in 5/41 lumpectomies or mastectomies but only corresponded to invasive cancer in one case. In two cases, the false positive wound bed fluorescence was associated with suture granuloma and mammary lobular hyperplasia, respectively. Accumulation of ICG in areas of inflammation is a known limitation of this imaging agent and both these conditions were associated with some degree of inflammation and possibly increased capillary permeability [25]. The residual fluorescence corresponding to normal muscle and linea alba may be due to ICG leakage from the tumor interstitium during specimen manipulation. Analogous results have been seen in a trial of ICG imaging in human breast cancer where specimen manipulation resulted in the expression of fluorescent fluid [44].

The current gold standard for sentinel lymph node mapping in human breast cancer is peritumoral injection of a radiotracer preoperatively and a blue dye perioperatively, then intraoperative lymphoscintigraphy and visualization of the colored lymphatics and lymph nodes [22,45]. However, NIR fluorescence imaging using ICG for sentinel lymph node mapping in breast cancer has been shown to be technically feasible [46] and has produced results similar to or better than current techniques [23,47]. All of these studies have used periareolar or peritumoral injections of ICG. One study describes the use of systemically administered ICG to evaluate human breast cancer margins, but lymph node imaging was not reported [44]. In this study we used systemically administered ICG to identify the primary tumor and visualize tumor margins. In addition, 4/7 lymph nodes in resected specimens fluoresced with systemically administered ICG and NIR imaging. Two of these fluorescent lymph nodes contained tumor metastases. The two remaining fluorescent nodes were tumor negative but draining malignant tumors. This limited data suggests that systemically administered NIR imaging agents could be used to identify sentinel lymph nodes and nodes containing metastatic disease. However, ICG lacks specificity to discriminate between node fluorescence due to metastatic disease and fluorophore drainage from the primary tumor. Molecularly targeted NIR fluorophores may be more appropriate for this objective.

There are several limitations to our study. Firstly, a large margin of grossly normal mammary tissue was taken around the tumor in these cases as this is the standard of veterinary care for this disease. This does not recapitulate BCS in patients where, in addition to clean surgical margins, goals include preservation of as much normal tissue as possible for the best cosmetic result. We had no tumor-positive margins in this study, and the amount of tissue resected may have also influenced the observed rate of residual wound bed fluorescence. Secondly, lymph nodes are not normally removed in canine lumpectomies (regional mastectomies) unless they are palpably enlarged or incorporated into a mastectomy involving one or both caudal mammary glands.

Despite these limitations, our results support the feasibility of using spontaneous canine mammary tumors as a model to evaluate new intraoperative NIR imaging agents and systems. Although ICG is an FDA approved compound and is broadly applicable across a range of solid tumors, it has a relatively low quantum yield and this study shows that it has poor specificity for malignant breast neoplasia. A breast cancer targeted imaging agent could potentially decrease non-specific dye accumulation, improve quantum yield, and allow for more reliable detection of small tumors and lymph node metastases. We have previously demonstrated that OTL 038, a folate receptor-targeted NIR agent, can identify spontaneously occurring lung cancers and lymph node metastases [48]. The folate alpha receptor is upregulated in some breast cancers, particularly triple negative breast cancer, which suggests OTL 038 may be an excellent targeted agent for imaging this human breast cancer subtype [49].

In conclusion, this study demonstrates that intraoperative imaging can identify canine mammary tumors, a spontaneous, large animal model of human breast cancer. Systemic administration of NIR contrast can identify both the tumor and sentinel lymph node, although improvements in specificity are required before human clinical application. This model could be used to study new targeted intraoperative NIR imaging agents that could improve detection of smaller tumors and metastatic lymph nodes.

## References

[1] Siegel RL, Miller KD, Jemal A. Cancer Statistics, 2017. CA Cancer J Clin. 2017. 10.3322/caac.21387 28055103

[2] Stang A, Kaab-Sanyal V, Hense HW, Becker N, Kuss O. Effect of mammography screening on surgical treatment for breast cancer: a nationwide analysis of hospitalization rates in Germany 2005–2009. Eur J Epidemiol. 2013;28(8):689–696. 10.1007/s10654-013-9816-9 23775424

[3] Early Breast Cancer Trialists' Collaborative Group. Effects of radiotherapy and surgery in early breast cancer. An overview of the randomized trials. N Engl J Med. 1995;333(22):1444–1455. 7477144 10.1056/NEJM199511303332202

[4] Fisher B, Anderson S, Bryant J, et al Twenty-year follow-up of a randomized trial comparing total mastectomy, lumpectomy, and lumpectomy plus irradiation for the treatment of invasive breast cancer. N Engl J Med. 2002;347(16):1233–1241. 10.1056/NEJMoa022152 12393820

[5] van Dongen JA, Voogd AC, Fentiman IS, et al Long-term results of a randomized trial comparing breast-conserving therapy with mastectomy: European Organization for Research and Treatment of Cancer 10801 trial. J Natl Cancer Inst. 2000;92(14):1143–1150. 10.1093/jnci/92.14.1143 10904087

[6] Bijker N, Peterse JL, Duchateau L, et al Risk factors for recurrence and metastasis after breast-conserving therapy for ductal carcinoma-in-situ: analysis of European Organization for Research and Treatment of Cancer Trial 10853. J Clin Oncol. 2001;19(8):2263–2271. 10.1200/JCO.2001.19.8.2263 11304780

[7] Weng EY, Juillard GJ, Parker RG, Chang HR, Gornbein JA. Outcomes and factors impacting local recurrence of ductal carcinoma in situ. Cancer. 2000;88(7):1643–1649. 10738223

[8] Jeevan R, Cromwell DA, Trivella M, Lawrence G, Kearins O, Pereira J, et al Reoperation rates after breast conserving surgery for breast cancer amoung women in England: retrospective study of hospital episode statistics. BMJ 2012;345:e4505 10.1136/bmj.e4505 22791786 PMC3395735

[9] McCahill LE, Single RM, Aiello Bowles EJ, et al Variability in reexcision following breast conservation surgery. JAMA. 2012;307(5):467–475. 10.1001/jama.2012.43 22298678

[10] Langhans L, Jensen MB, Talman MM, Vejborg I, Kroman N, Tvedskov TF. Reoperation rates in ductal carcinoma in situ vs invasive breast cancer after wire-guided breast-conserving surgery. JAMA Surg. 2017;152(4):378–384. 10.1001/jamasurg.2016.4751 28002557 PMC5470426

[11] Schulman AM, Mirrielees JA, Leverson G, Landercasper J, Greenberg C, Wilke LG. Reexcision surgery for breast cancer: An analysis of the American Society of Breast Surgeons (ASBrS) Mastery(SM) database following the SSO-ASTRO "No Ink on Tumor" Guidelines. Ann Surg Oncol. 2017;24(1):52–58. 10.1245/s10434-016-5516-5 27581607

[12] Wilke LG, Czechura T, Wang C, Lapin B, Liederbach E, Winchester DP, et al Repeat surgery after breast conservation for the treatment of stage 0 to II breast carcinoma: a report from the National Cancer Data Base, 2004–2010. JAMA Surg. 2014;149(12):1296–1305. 10.1001/jamasurg.2014.926 25390819

[13] Baliski CR, Pataky RE. Influence of the SSO/ASTRO margin reexcision guidelines on costs associated with breast-conserving surgery. Ann Surg Oncol. 2017;24(3):632–637. 10.1245/s10434-016-5678-1 27882469

[14] Olsen MA, Nickel KB, Margenthaler JA, Wallace AE, Mines D, Miller JP, et al Increased risk of surgical site iunfection among breast-conserving surgery re-excisions. Ann Surg Oncol. 2015;22(6):2003–2009. 10.1245/s10434-014-4200-x 25358666 PMC4693603

[15] Moore MM, Whitney LA, Cerilli L, Imbrie JZ, Bunch M, Simpson VB, et al Intraoperative ultrasound is associated with clear lumpectomy margins for palpable infiltrating ductal breast cancer. *Ann Surg*. 2001;233(6):761–768. 10.1097/00000658-200106000-00005 11371734 PMC1421318

[16] Krekel NM, Haloua MH, Lopes Cardozo AM, deWit RH, Bosch AM, de Widt-Levert LM, et al Intraoperative ultrasound guidance for palpable breast cancer excision (COBALT trial): a multicentre, randomised controlled trial. *Lancet Oncol*. 2013;14(1):48–54. 10.1016/S1470-2045(12)70527-2 23218662

[17] Camp MS, Valero MG, Opara N, Benabou K, Cutone L, Caragacianu D, et al Intraoperative digital specimen mammography: a significant improvement in operative efficiency. *Am J Surg*. 2013;206(4):526–529. 10.1016/j.amjsurg.2013.01.046 23806823

[18] Gray RJ, Salud C, Nguyen K, Dauway E, Friedland J, Berman C, et al Randomized prospective evaluation of a novel technique for biopsy or lumpectomy of nonpalpable breast lesions: radioactive seed versus wire localization. *Ann Surg Oncol*. 2001;8(9):711–715. 10.1007/s10434-001-0711-3 11597011

[19] Schnabel F, Boolbol SK, Gittleman M, Karni T, Tafra L, Feldman S, et al A randomized prospective study of lumpectomy margin assessment with use of MarginProbe in patients with nonpalpable breast malignancies. *Ann Surg Oncol*. 2014;21(5):1589–1595. 10.1245/s10434-014-3602-0 24595800 PMC3975090

[20] Esbona K, Li Z, Wilke LG. Intraoperative imprint cytology and frozen section pathology for margin assessment in breast conservation surgery: a systematic review. *Ann Surg Oncol*. 2012;19(10):3236–3245. 10.1245/s10434-012-2492-2 22847119 PMC4247998

[21] de Boer E, Harlaar NJ, Taruttis A, Nagengast WB, Rosenthal EL, Ntziachristos V, et al Optical innovations in surgery. *Br J Surg*. 2015;102(2):e56–72. 10.1002/bjs.9713 25627136

[22] Goyal A, Newcombe RG, Chhabra A, Mansel RE, Group AT. Factors affecting failed localisation and false-negative rates of sentinel node biopsy in breast cancer—results of the ALMANAC validation phase. *Breast Cancer Res Treat*. 2006;99(2):203–208. 10.1007/s10549-006-9192-1 16541308

[23] Troyan SL, Kianzad V, Gibbs-Strauss SL, Gioux S, Matsui A, Oketokoun R, et al The FLARE intraoperative near-infrared fluorescence imaging system: a first-in-human clinical trial in breast cancer sentinel lymph node mapping. *Ann Surg Oncol*. 2009;16(10):2943–2952. 10.1245/s10434-009-0594-2 19582506 PMC2772055

[24] Madajewski B, Judy BF, Mouchli A, Kapoor V, Holt DE, Wand MD, et al Intraoperative near-infrared imaging of surgical wounds after tumor resections can detect residual disease. *Clin Cancer Res*. 2012;18(20):5741–5751. 10.1158/1078-0432.CCR-12-1188 22932668 PMC3482441

[25] Holt D, Okusanya O, Judy R, Venegas O, Jiang J, DeJesus E, et al Intraoperative near-infrared imaging can distinguish cancer from normal tissue but not inflammation. *PLoS One*. 2014;9(7):e103342 10.1371/journal.pone.0103342 25072388 PMC4114746

[26] Holt D, Parthasarathy AB, Okusanya O, Keating J, Venegas O, Deshpande C, et al Intraoperative near-infrared fluorescence imaging and spectroscopy identifies residual tumor cells in wounds. *J Biomed Opt*. 2015;20(7):76002 10.1117/1.JBO.20.7.076002 26160347 PMC4497968

[27] Queiroga FL, Raposo T, Carvalho MI, Prada J, Pires I. Canine mammary tumours as a model to study human breast cancer: most recent findings. *In Vivo*. 2011;25(3):455–465. 21576423

[28] Sorenmo KU, Rasotto R, Zappulli V, Goldschmidt MH. Development, anatomy, histology, lymphatic drainage, clinical features, and cell differentiation markers of canine mammary gland neoplasms. *Vet Pathol*. 2011;48(1):85–97. 10.1177/0300985810389480 21147765

[29] Nguyen F, Pena L, Ibisch C, Loussouarn D, Gama A, Rieder N, et al Canine invasive mammary carcinomas as models of human breast cancer. Part 1: natural history and prognostic factors. *Breast Cancer Res Treat*. 2018;167(3):635–648. 10.1007/s10549-017-4548-2 29086231 PMC5807494

[30] Abadie J, Nguyen F, Loussouarn D, Pena L, Gama A, Rieder N, et al Canine invasive mammary carcinomas as models of human breast cancer. Part 2: immunophenotypes and prognostic significance. *Breast Cancer Res Treat*. 2018;167(2):459–468. 10.1007/s10549-017-4542-8 29063312 PMC5790838

[31] Liu D, Xiong H, Ellis AE, Northrop NC, Rodriguez CO Jr, O'Regan RO, et al Molecular homology and difference between spontaneous canine mammary cancer and human breast cancer. *Cancer Res*. 2014;74(18):5045–5056. 10.1158/0008-5472.CAN-14-0392 25082814 PMC4167563

[32] Reynolds JS, Troy TL, Mayer RH, Thompson AB, Waters DJ, Cornell KK, et al Imaging of spontaneous canine mammary tumors using fluorescent contrast agents. Photochem Photobiol 1999; 70(1):87–94. 10420847

[33] Gurfinkel M, Thompson AB, Ralston W, Troy TL, Moore AL, Moore TA, et al Pharmacokinetics of ICG and HPPH-car for the detection of normal and tumor tissue using fluorescence, near-infrared reflectance imaging: A case study. Photochem Photobiol 2000; 72(1):94–102. 10911733 10.1562/0031-8655(2000)072<0094:poiahc>2.0.co;2

[34] Fidel J, Kennedy KC, Dernell WS, Hansen S, Wiss V, Stroud MR, et al Preclinical validation of the utility of BLZ-100 in providing fluorescence contrast for imaging canine spontaneous solid tumors. Cancer Res 2015; 75(20):4283–4291. 10.1158/0008-5472.CAN-15-0471 26471914 PMC4610180

[35] Valejo FAM, Tiezzi DG, Mandarano LRM, de Sousa CB, de Andrade JM. Volume of breast tissue excised during breast-conserving surgery in patients undergoing preoperative systemic therapy. Rev Bras Ginecol Obstet 2013; 35 (3):221–225. 23843120 10.1590/s0100-72032013000500006

[36] Matsumura Y, Maeda H. A new concept for macromolecular therapeutics in cancer chemotherapy: mechanism of tumoritropic accumulation of proteins and the antitumor agent smancs. Cancer Res 1986;46(12 Pt 1):6387–6392. 2946403

[37] Maeda H, Matsumura Y. EPR effect based drug design and clinical outlook for enhanced cancer chemotherapy. Adv Drug Deliv Rev 2011;63(3):129–130. 10.1016/j.addr.2010.05.001 20457195

[38] Heneweer C, Holland JP, Divilov V, Carlin S, Lewis JS. Magnitude of enhanced permeability and retention effect in tumors with different phenotypes: 89Zr-albumin as a model system. J Nucl Med 2011;52(4):625–633. 10.2967/jnumed.110.083998 21421727 PMC3902086

[39] Kosaka N, Mitsunaga M, Longmire MR, Choyke PL, Kobayashi H. Near infrared fluorescence-guided real-time endoscopic detection of peritoneal ovarian cancer nodules using intravenously injected indocyanine green. Int J Cancer 2011;129(7):1671–1677. 10.1002/ijc.26113 21469142 PMC3145021

[40] Bos R, Zhong H, Hanrahan CF, Mommers ECM, Semenza GL, Pinedo HM, et al Levels of hypoxia-inducible factor-1α during breast carcinogenesis. J Natl Cancer Ins 2011; 93; (4):309–314. 10.1093/jnci/93.4.309 11181778

[41] KleinJan GH, Bunschoten A, van den Berg NS, Valdes Olmos RA, Klop WMC, Horenblas S, et al Fluorescence guided surgery and tracer-dose, fact or fiction? Eur J Nucl Med Mol Imaging 2016;43(10):1857–1867. 10.1007/s00259-016-3372-y 27020580 PMC4969335

[42] Graham JC, Myers RK. The prognostic significance of angiogenesis in canine mammary tumors. J Vet Int Med 1999;13:416–418. 10.1892/0891-6640(1999)013<0416:tpsoai>2.3.co;2 10499723

[43] Sleeckx N, Van Brantegem L, Van den Eynden G, Fransen E, Casteleyn C, Van Cruchten S, et al Angiogenesis in canine mammary tumours: a morphometric and prognostic study. J Comp Pathol. 2014;150(2–3):175–83. 10.1016/j.jcpa.2013.09.005 24231306

[44] Keating J, Tchou J, Okusanya O, Fisher C, Batiste R, Jiang J, et al Identification of breast cancer margins using intraoperative near-infrared imaging. J Surg Oncol 2016;113(5):508–514. 26843131 10.1002/jso.24167 PMC11156255

[45] Verbeek FPR, Troyan SL, Mieog JSD, Liefers G-J, Moffitt LA, Rosenberg M, et al Near-infrared fluorescence sentinel lymph node mapping in breast cancer: a multicenter experience. Breast Cancer Res Treat 2014; 143 (2):333–342. 10.1007/s10549-013-2802-9 24337507 PMC3899688

[46] Chi C, Ye J, Ding H, He D, Huang W, Zhang G-J, et al Use of indocyanine green for detecting the sentinel lymph node in breast cancer patients: From preclinical evaluation to clinical validation. PLOS ONE 2013; 8(12):e83927 10.1371/journal.pone.0083927 24358319 PMC3865279

[47] Samorani D, Fogacci T, Panzini I, Frisoni G, Accardi FG, Ricci M, et al The use of indocyanine green to detect sentinel nodes in breast cancer: A prospective study. Eur J Surg Oncol 2015; 41: 64–70. 10.1016/j.ejso.2014.10.047 25468752

[48] Keating JJ, Runge JJ, Singhal S, Nims S, Venegas O, Durham AC, et al Intraoperative near-infrared fluorescence imaging targeting folate receptors identifies lung cancer in a large-animal model. Cancer 2017;123(6):1051–1060. 10.1002/cncr.30419 28263385 PMC5341137

[49] Boogerd LS, Boonstra MC, Beck AJ, Charehbili A, Hoogstins CES, Prevoo HAMJ, et al Concordance of folate receptor-alpha expression between biopsy, primary tumor and metastasis in breast cancer and lung cancer patients. Oncotarget 2016;7(14):17442–17454. 10.18632/oncotarget.7856 26943581 PMC4951224

